# The Transactions of the Provincial Medical and Surgical Association

**Published:** 1837-10

**Authors:** 


### Art. XIII.

*The Transactions of the Provincial Medical and Surgical Association.* Vol. V.—*London*, 1837. 8vo. pp. 527. With Plates.

Like its predecessors, the present volume continues to evince the activity and zeal of the members of the Association; we think, however, it is, on the whole, inferior to some of those which have already appeared. The Annual Address, instituted for the purpose of taking a retrospective view of the advancement of medical science during the previous year, was delivered by J. G. Crosse, Esq., of Norwich. In a previous number, we have alluded to this elaborate and valuable paper, containing, as it does, and especially in that part of it which is devoted to surgical science, a minute detail of all that had been done for the improvement of our profession during the preceding twelve months. It partakes too much of the nature of an analytical review to admit of being again reviewed. We shall also pass over the papers on *Medical Topography* at present, as we have prepared a separate article on all those which have appeared in the Transactions, and which we hope to give in our next Number.

The third division of the Volume consists of Essays and Cases, some of which we shall shortly notice, particularly such as are of practical value. The account which Mr. Jeaffreson has given of *the successful removal of an Ovarian Tumour*, is interesting. His object was to operate "as soon as the sac was sufficiently distended to press firmly on the parietes of the abdomen, and before adhesion had taken place." A case, presenting the above conditions was operated upon in the following manner: "An incision of between ten and twelve lines was made in the linea alba, midway between the navel and pubes, and, having thus carefully exposed the sac, about twelve pints of clear serum were evacuated by a trocar. During the flow of the serum, a portion of the sac was secured in the gripe of a forceps, to prevent its receding, and the sac was afterwards extracted entire from the cavity of the abdomen, together with another sac, containing two ounces of fluid, and indeed, the whole ovary, having only to cut through a slight reflexion of the peritoneum and ovarian ligament, which, with the exception of a small portion of the fimbriated extremity of the fallopian tube, are the only natural attachments of the ovary to the uterus. But, as this part was the medium of vascular supply to the sac, and the vessels on the surface of the sac were unusually large, it was included in a ligature previous to returning it into the cavity of the abdomen: the ends of the ligature were cut off close to the knot." The result of this operation was perfectly successful.

Mr. Jeaffreson adds, that the objection which has been urged against early tapping in ovarian dropsy, from the fluid being sometimes contained in several cysts, appears by this mode of operating to be completely avoided, as it gives the opportunity of tapping them in succession, if necessary, and, at all events, of removing the whole of the disease. But it must be borne in mind, that the operation must be performed before adhesion has taken place between the sac and the adjacent viscera.

Mr. Hunt endeavours to show, that the *superior oblique and abductor muscles of both eyes*, supplied as they are by separate pairs of nerves, cannot act at the same time; whilst those muscles which derive their influence from the third pair generally act consentaneously in both eyes, and cannot, under ordinary circumstances, act independently of each other. The final cause of such nervous distribution, it will be seen, on reflecting upon the actions of the various muscles of the eye, is, that the globe may always be directed towards an object in such a manner as shall ensure the correspondence of the axis of each eye, and thus the perception of a single image by the brain.

Dr. Dick has concluded his remarks on the *Unity of Organic Structure*, which, as containing an outline of various observations and speculations on the correspondence of organs and systems in different gradations of animals during their various degree of development, are well worthy of perusal; but which we cannot further notice in this place.

Two cases of *Encysted Dropsy of the Thyroid Gland* are related by Dr. Selwyn. One of these was cured by a seton, without any untoward accident; the other was treated with similar success by the destruction of a portion of the cyst by potassa fusa.

Mr. Poyser has recorded some *Cases and Dissections chiefly in reference to the Uncertainty of Diagnosis.* A faithful chronicle of practical errors would constitute a work of far more value than those which record the miracles of medicine with which we not unfrequently meet; and we trust that in this respect Mr. Poyser will not be without his followers. The present cases are illustrative of the obscurity attending some cases of perforation of the stomach, and of the sensation of fluctuation communicated to the finger by fungous tumours, leading occasionally to an operation for the removal of fluid which it may generally be expedient to postpone.

We can scarcely agree with Mr. Hamerton in his inference respecting *a case of Tetanus, successfully treated by Carbonate of Iron.* During the course of the attack, the patient consumed a pound and a half of carbonate of iron, and he recovered; but, together with the iron, a variety of remedial means was employed, so that, whether his recovery was post or propter unguentum lyttæ, bleeding, turpentine per os et anum, liquorice, blisters, &c., individually or collectively, or iron, it would be difficult to determine. As an instance, however, of the safety of taking a pound and a half of carbonate of iron in a fortnight, (a practice which, we trust, is falling somewhat into disuse,) it is not without its value.

Dr. J. Shapter has given an account of a *curious case of incapacity to articulate*, and of *loss of memory of languages.* The individual was highly educated and a "perfect master of several languages." His illness followed great mental anxiety, fatigue, and deficient nourishment; and was characterized by great irritability and cerebral excitement, which would ill bear depleting remedies. At first there was a "total loss of memory for languages." In the later progress of the case, "he could recollect *portions* of a few words, and, after repeated trials, could write some of the short ones correctly, without the assistance of a dictionary." The following is a copy of a note, written after much labour. "Grandfather is in apossema in head—in a cure disease in forty-one year—he after dieth blood in blood. I in I an *apossema* in head in a case diseas—not in blood—young my brother nine in a case diseas of encephalon died—fifteen no can speak—(cephalic snuff) (I can not speak, non read, non write." At the time when Dr. Shapter lost sight of his patient,

(seventeen months after the commencement of his illness,) he was somewhat improved; but "his powers of reading are soon exhausted, and he has for the most part lost the faculty of properly arranging and constructing his sentences, and is now almost totally incapable of articulating with correctness the few words he has with difficulty reacquired." Dr. Shapter hazards the opinion, that the proximate cause of the malady may have been the bursting of a blood-vessel at the base of the brain, or the superior portion of the spinal column; and that some coagulum had formed near that centre whence arise the glosso-pharyngeal and lingual nerves.

Mr. Salter's *case of Malignant Tumour within the Abdomen* is very interesting, although it is to be lamented that circumstances prevented an examination after death. To have traced, however, during life, and to have recorded the progress of such a disease, is valuable, independent of the knowledge which might have been obtained after death. We entirely concur with Mr. Salter in his opinion as to the malignity of the disease, and as to the mode in which the intestine became implicated therein.

Dr. Norris has given an account of a *Case of Death from Diaphragmatic Hernia.* It is also another instance of transposition of viscera. In this instance, some portions of the abdominal viscera, long resident within the left cavity of the thorax, had become strangulated, the communication between the cavities being an aperture in the left portion of the diaphragm, near the vertebræ, more than an inch in diameter. The symptoms (those of obstruction) were such as could only lead to a suspicion of the nature of the disease during life.

Dr. Walker's *Cases of Rheumatic Metastasis* are interesting illustrations of a fact with which the profession is now so familiar that we need not repeat them in this place.

Under the title "*Effects of Chronic Pleuritis,*" Mr. Windsor has related, first, an instance of empyema communicating with an abscess in the back; next, a case of a child aged seven years, in which the removal of a fluid effusion into the left pleura, sufficient to displace the heart considerably towards the right side, appeared to be facilitated by a combination of blue pill, digitalis, squill, and elaterium, and a demulcent mixture. The last case is one of a fatal termination of paracentesis thoracis.

Part the Fourth consists of *Reports of the Birmingham Eye Infirmary,* by R. Middlemore, Esq., of the *Birmingham Dispensary,* by Dr. T. O. Ward, and of the *Worcester Infirmary.* The two former reports are characterized by the same care as those of previous volumes: we can, however, only refer to them in this place.

As an appendix to the Volume are added the observations by Messrs. Rumsey and Ceely, "*on the present condition of Medical Relief for the Sick Paupers, with recommendations for an altered and improved system.*" It is probable that few of our readers who feel themselves interested in this subject, have not had an opportunity of reading this valuable pamphlet. On some future occasion we hope to go into the consideration of the whole subject.

